# Genetic and Functional Variants Analysis of the *GATA6* Gene Promoter in Acute Myocardial Infarction

**DOI:** 10.3389/fgene.2019.01100

**Published:** 2019-11-06

**Authors:** Zhaoqing Sun, Shuchao Pang, Yinghua Cui, Bo Yan

**Affiliations:** ^1^Cheeloo College of Medicine, Shandong University, Jinan, China; ^2^Shandong Provincial Key Laboratory of Cardiac Disease Diagnosis and Treatment, Affiliated Hospital of Jining Medical University, Jining Medical University, Jining, China; ^3^Division of Cardiology, Affiliated Hospital of Jining Medical University, Jining Medical University, Jining, China; ^4^The Center for Molecular Genetics of Cardiovascular Diseases, Affiliated Hospital of Jining Medical University, Jining Medical University, Jining, China; ^5^Shandong Provincial Sino-US Cooperation Research Center for Translational Medicine, Affiliated Hospital of Jining Medical University, Jining Medical University, Jining, China

**Keywords:** acute myocardial infarction, GATA binding protein 6, promoter, single nucleotide polymorphisms, genetic variants, gene expression regulation

## Abstract

**Background:** Acute myocardial infarction (AMI) which is a specific type of coronary artery disease (CAD), is caused by the combination of genetic factors and acquired environment. Although some common genetic variations have been recorded to contribute to the development of CAD and AMI, more genetic factors and potential molecular mechanisms remain largely unknown. The *GATA6* gene is expressed in the heart during embryogenesis and is also detected in vascular smooth muscle cells (VSMCs), different human primary endothelial cells (ECs), and vascular ECs in mice. To date, no studies have directly linked *GATA6* gene with regulation of the CAD.

**Methods:** In this study, we used a case-control study to investigate and analyze the genetic variations and functional variations of the *GATA6* gene promoter region in AMI patients and controls. A variety of statistical analysis methods were utilized to analyze the association of single nucleotide polymorphisms (SNPs) with AMI. Functional analysis of DNA sequence variants (DSVs) was performed using a dual luciferase reporter assay. In vitro, electrophoretic mobility shift assay (EMSA) was selected to examine DNA-protein interactions.

**Results:** A total of 705 subjects were enrolled in the study. Ten DSVs were found in AMI patients (n = 352) and controls (n = 353), including seven SNPs. One novel heterozygous DSV, (g.22168409 A > G), and two SNPs, [g.22168362 C > A(rs1416421760) and g.22168521 G > T(rs1445501474)], were reported in three AMI patients, which were not found in controls. The relevant statistical analysis, including allele and genotype frequencies between AMI patients and controls, five genetic models, linkage disequilibrium (LD) and haplotype analysis, and SNP–SNP interactions, suggested no statistical significance (*P* > 0.05). The transcriptional activity of *GATA6* gene promoter was significantly increased by the DSV (g.22168409 A > G) and SNP [g.22168362 C > A(rs1416421760)]. The EMSA revealed that the DSV (g.22168409 A > G) and SNP [g.22168362 C > A(rs1416421760)] evidently influenced the binding of transcription factors.

**Conclusion:** In conclusion, the DSV (g.22168409 A > G) and SNP [g.22168362 C > A(rs1416421760)] may increase GATA6 levels in both HEK-293 and H9c2 cell lines by affecting the binding of transcription factors. Whether the two variants identified in the *GATA6* gene promoter can promote the development and progression of human AMI by altering GATA6 levels still requires further studies to verify.

## Introduction

Coronary artery disease (CAD), the most common cardiovascular disease in humans, is a major cause of death and disability in both developed and developing nations. In fact, CAD, including acute myocardial infarction (AMI) which is a specific type of CAD, is caused by the combination of genetic factors and acquired environment. So far, we have widely recognized that the top risk factors related to CAD include aging, dyslipidemia, hypertension, smoking, diabetes, metabolic syndrome, hyperhomocysteinemia, and C-reactive protein (Mack and Gopal, 2014). Lipid metabolism disorders and inflammation play a key role in the development and progression of atherosclerosis (AS) and its complications, particularly plaque rupture and subsequent AMI (Connelly et al., 2016; Shapiro and Fazio, 2016). In the past few years, large-scale population researches and rapid development of genetic technology have revolutionized our understanding of the genetic basis of CAD. Many genome-wide association studies (GWAS) have identified a large number of genetic loci, suggesting that common genetic variations contribute to the development of CAD and AMI (Björkegren et al., 2015; Assimes and Roberts, 2016; McPherson and Tybjaerg-Hansen, 2016). However, among the identified susceptible alleles, most are common in the population, could only explain <10% of the genetic variance in CAD (Lettre, 2014). These studies suggest that in addition to the high-order interaction between genes and environmental factors, there may be some rare susceptibility alleles *in vivo*, and/or other unknown molecular biology/genetic mechanisms that contribute to the occurrence of CAD and AMI. Based on previous epidemiological studies, we found that the incidence of cardiovascular events and mortality was significantly higher in adults with congenital heart disease (CHD) than in the general population, with myocardial infarction (MI) being the leading cause of death (Tutarel et al., 2013; Sieweke et al., 2016; Fedchenko et al., 2017; Olsen et al., 2017). Therefore, dysregulation of cardiac developmental genes may contribute to the pathogenesis of CAD and further genetic analysis is necessary.

The GATA family of transcription factors is a highly conserved DNA binding domain containing two zinc finger structures (cys-x2-cys-x17-cys-x2-cys), which preferentially bind to a 5’-(A/T)GATA(A/G)-3’ motif within the regulatory region of target genes (Oka et al., 2007). In vertebrates, the GATA family is composed of six members (GATA1∼GATA6) and plays a key role in regulating the growth, differentiation, and survival of various cells and maintaining the function of the body (Patient and McGhee, 2002). Members of the GATA transcription factor are not only prominently expressed in hematopoietic cells, but are also widely expressed in the developing heart and the dermal lineage including the gastrointestinal tract (Molkentin, 2000). Mutations in certain gene bases in the GATA family are associated with human developmental disorders such as anemia, hypoparathyroidism, deafness and infertility, and kidney and heart defects (Tremblay et al., 2018). In previous reports, a large number of studies have confirmed that *GATA6* gene is expressed during cardiac development. At present, CHD associated with *GATA6* gene mutations include bicuspid aortic valve (BAV), ventricular septal defect (VSD), patent ductus arteriosus (PDA), atrial septal defect (ASD), persistent truncus arteriosus (PTA), and tetralogy of Fallot (TOF) (Kodo et al., 2009; Lin et al., 2010; Maitra et al., 2010; Kodo et al., 2012; Wang et al., 2012; Zheng et al., 2012; Bui et al., 2013; Huang et al., 2013; Li et al., 2014; Gharibeh et al., 2018; Xu et al., 2018). In addition, sequence variations in the splicing sites and coding regions of *GATA6* have also been reported in other cardiovascular diseases such as dilated cardiomyopathy (DCM) (van Berlo et al., 2010; Xu et al., 2014) and cardiac conduction system (CCS) including atrial fibrillation (AF) (Li et al., 2012; Yang et al., 2012; Liu et al., 2015; Nemer and Gharibeh, 2015; Tucker et al., 2017).

However, it has been reported that the *GATA6* gene is expressed in the heart during embryogenesis and is also detected in vascular smooth muscle cells (VSMCs), different human primary endothelial cells (ECs), and vascular ECs in mice (Wada et al., 2000; Froese et al., 2011). In the GATA family, only *GATA6* is expressed in VSMCs and plays a role in maintaining the differentiation phenotype of VSMCs (Wada et al., 2000). In terms of mechanism, *GATA6* plays an important role in promoting EC function and survival by inhibiting the autocrine release of TGF-1 and TGF-2, both of which act as angiogenesis inhibitors (Froese et al., 2011). Based on GWAS studies on CAD and MI, we can understand the presence of changes in the differentiation of VSMCs in the pathogenesis of CAD (Motterle et al., 2012; Nurnberg et al., 2015). Of note, primary human umbilical vein endothelial cells (HUVECs) express high levels of GATA6, which regulates the expression of the vascular cell adhesion molecule-1 (VCAM-1) in these cells (Umetani et al., 2001). Specifically, VCAM-1 plays a central role in the initiation of atherosclerosis by mediating firm adhesion of activated monocytes to the arterial wall *via* binding with α4β1 integrin (Cybulsky et al., 2001; Ley et al., 2007). AS is the main cause of CAD, in which lipid metabolism disorders and inflammation are the basis of AS lesions. When the arterial wall thickens and the vascular lumen is narrowed to block the arterial lumen, the tissue or organ supplied by the artery will be ischemic or necrotic (Cahill and Redmond, 2016). Simultaneously, it is the principal pathologic condition underlying CAD leading to heart attack. Although the intracellular content of GATA6 has been reported to be involved in the inflammatory response and has a linear relationship with the expression of CD14/CD16 (Ruiz-Alcaraz et al., 2018), to date, no studies have directly linked *GATA6* to the development of CAD. DNA sequence variants (DSVs) and single nucleotide polymorphisms (SNPs) within the *GATA6* gene promoter which are founded in AMI patients have not been studied and reported. Therefore, we sought to determine whether genetic mutations in the *GATA6* promoter region are involved in the formation and progression of AMI by affecting transcriptional activity.

In this innovative study, we performed genetic variations and functional variations analysis of human *GATA6* gene promoters in a large number of AMI patients and healthy controls.

## Materials and Methods

### AMI Patients and Healthy Controls

A total of 352 patients with AMI who were enrolled in the study were recruited from the Cardiac Intensive Care Unit of the Affiliated Hospital of Jining Medical College, Shandong Province, China from December 2015 to February 2017. There were 250 males and 102 females, ranging in age from 28 to 94, with an average age of 63.50 years. Patients with AMI were diagnosed with medical records, physical examination, electrocardiogram, and three-dimensional echocardiography. At the same time, we recruited 353 patients who had no family history of CAD and who had the same ethnicity and no blood relationship with AMI patients. The controls included 225 males and 128 females, ranging in age from 19 to 83 years with an average age of 45.72 years. The research protocol was approved by the Humanities and Ethics Committee of the Affiliated Hospital of Jining Medical University and was conducted in accordance with the principles of the Helsinki Declaration. The study received informed consent from all participants.

### Direct DNA Sequencing of the *GATA6* Gene Promoter

From the Genebank database, about 2,000 bases upstream of the transcription start point of the *GATA6* gene (NCBI: NC_000018.10) were selected for analysis and PCR primers for the *GATA6* gene promoter were designed based on the sequence. The final length was 1,315 bp (-1240 to +75 bp from the transcription start site). In order to facilitate the acquisition of all target fragments, we used two overlapping DNA fragments 695bp (∼1240∼-514bp) and 663bp (-588∼ + 75bp) for RCR amplification. The information for all primers has been clearly summarized in **Table 1**. The PCR products were bi-directionally sequenced with 3500 Genetic Analyzer (Applied Biosystems, Foster City, CA, USA). DNA sequences were aligned and compared with the wild-type sequence of the *GATA6* gene promoter.

**Table 1:** PCR primers for the human *GATA6* gene promoter^a^.

Primers	Sequences	Location	Products
Sequencing			
*GATA6*-F1	5’-ACCAGAGCCTAAACGCTTTC-3’	22168197	695bp
*GATA6*-R1	5’-ACCCTATCTCGGGATGCTAC-3’	22168891	
*GATA6*-F2	5’-CCGAAACCACCACGACCTGAG-3’	22168849	663bp
*GATA6*-R2	5’-TGGGCTCCTGATTGGACTCACC-3’	22169511	
Functioning			
*GATA6*-F	5’-(KpnI)-ACGCCTCTTGTCCTAAAGTCTC-3’	22168318	1173bp
*GATA6*-R	5’-(HindIII)-CGAGCCCTAAACAAACAGC-3’	22169490	

^a^PCR primers were designed based on the genomic DNA sequence of the human GATA6 gene. (NC_000018.10). The transcription start site (+1) is at position of 22169437.

### Functional Analysis of the DSVs by Dual-Luciferase Reporter Assay

By preliminary screening of the two PCR fragments, we further designed a PCR primer with a length of 1,173 bp (−1119 ∼ +54) in order to amplify the fragment containing the variant sites and the wild-type fragment in the promoter region of *GATA6* gene. In addition, we added a Kpn I site to the upper end of the *GATA6* forward primer and added the Hind III site to the *GATA6* reverse primer (**Table 1**). Expression constructs were generated by subcloning PCR products into Kpn I and Hind III sites of a reporter vector-pGL3-basic, expressing luciferase gene. We selected two cell lines including human embryonic kidney cells (HEK-293) and rat cardiomyocytes (H9c2) to detect the transcriptional activity of the *GATA6* gene promoter. We transiently transfected 1.5 ug of the designated expression construct into a 6-well plate in which HEK-293 cells were grown. In addition, 2.0 ug of the designated expression construct was also transiently transfected into 6-well plates grown with H9c2. Fresh medium was replaced after 5 h. Transfected cells were harvested after transfection for a specific time (36 h for HEK-293 and 48 h for H9c2). Expression constructs expressing the Renilla luciferase gene (pRL-TK) were used as internal controls in cells of HEK-293 (10 ng) and H9c2 (20 ng), respectively. At the same time, the empty vector pGL3-basic was used as a negative control. Luciferases activities were measured using dual-luciferase reporter assay system on a Glomax 20/20 luminometer (Promega, Madison, WI, USA). The transcriptional activities of the *GATA6* gene promoters were represented as ratios of luciferase over renilla luciferase activities. Transcriptional activity of the wild-type *GATA6* gene promoter was designed as 100%. All the experiments were repeated at least three times independently, in triplicate.

### Nuclear Extracts Preparation and Electrophoretic Mobility Shift Assay (EMSA)

Nuclear extracts were obtained from cultured HEK-293 and H9c2 cells using NE-PER^®^ nuclear and cytoplasmic extraction reagents (Thermo Scientific, Rockford, IL). The protein concentration of the nuclear extract was determined by Bio-Rad Protein Assay Reagent and stored at -80°C until use. The interaction between the DNA fragment of the *GATA6* gene promoter and nuclear protein was explored using EMSA and experiments were performed according to the protocol suggested by the LightShift^®^ Chemiluminescent EMSA kit (Thermo Scientific). The double-stranded biotinylated oligonucleotides were based on the wild-type and DSVs in the *GATA6* gene promoter. In order to completely bind the DNA and the protein, the reaction was mixed for 20 min at room temperature with an equal amount of biotinylated oligonucleotide and nuclear extract (3.0 µg). The mixture of DNA and protein was gently mixed with 5 ul of loading buffer and loaded onto a 0.5 x TBE natural 6% polyacrylamide gel. The voltage was set to 100 V at room temperature for 50 min. It was then transferred to a nylon membrane (Thermo Scientific) at 380 mA for 35 min in a low temperature environment. The oligonucleotides were cross-linked to the membrane using a UV Strata linker 1800 (Strata gene, Santa Clara, CA). Finally, biotinylated oligonucleotides were detected by chemiluminescence.

### Statistical Analysis

The quantitative data were represented as mean ± standard deviation (SD) or mean ± standard error of mean (SEM) and compared by a standard Student’s t-test. The qualitative data of AMI patients and controls and the distribution of DSVs in the *GATA6* gene promoter were analyzed and compared by chi-square test. Each SNP frequency in the AMI and control subjects was tested for deviation from Hardy–Weinberg equilibrium (HWE) by the Fisher’s test. Pearson chi‐squared test was performed to evaluate the significant differences on allele and genotype frequencies between AMI patients and controls. Odds ratio (OR) values and 95% confidence intervals (CIs) measured risk allele effect size using unconditional logistic regression analysis. Statistical analysis of the above data was done using Microsoft Excel and SPSS 22.0 statistical software packages. Five genetic models (codominant, dominant, over‐dominant, recessive, and log-additive) with an adjustment of age and sex were analyzed the association with AMI by the web‐based software SNP-Stats (https://www.snpstats.net/start.htm). HaploView software package (version 4.2) and the SHE-sis software platform (http://analysis.bio-x.cn/SHEsisMain.htm) were used to conduct linkage disequilibrium (LD) analysis and haplotype‐based associations. The generalized multi-factor dimensionality reduction (GMDR) software package (version 0.9) was used to explore SNP-SNP interactions and the best model with maximum cross-validation consistency was chosen. Results of the GMDR analysis were verified by traditional statistical methods. A statistical power was computed by a web browser program, Genetic Association Study Power Calculator (http://csg.sph.umich.edu/abecasis/gas_power_calculator/index.html). *P* < 0.05 was considered to be statistically significant.

## Results

### Characteristics of the Participants

This study involved 705 subjects, including 352 AMI patients and 353 healthy controls. The basic clinical data of the two groups were shown in **Table 2**. By comparing the clinical data of the two groups, we found that there were significantly more males than females in both the AMI and control groups. The mean age, blood pressure, blood glucose, smoking rate, and low-density lipoprotein cholesterol (LDL-C) levels in patients with AMI were significantly higher than those in control groups, and their high-density lipoprotein cholesterol (HDL-C) levels were lower than controls (P < 0.05), which were consistent with our traditional cognitive high risk factors for CAD. However, the level of triglyceride (TG) in the two groups was *P* > 0.05, which may be due to the use of statins in patients with AMI.

**Table 2:** Comparison of clinical data between AMI and controls in this study.

Variable^b^	AMI(n = 352)	Controls(n = 353)	*P* value
Sex (M/F)	250/102	225/128	0.039
Age (Y)	63.50 ± 12.20	45.72 ± 12.86	0.000
BMI (kg/m^2^)	24.89 ± 3.70	25.51 ± 3.69	0.031
Hypertension (%)	163 (46.3)	86 (24.4)	0.000
Diabetes (%)	78 (22.2)	26 (7.4)	0.000
Smoker (%)	194 (55.1)	56 (15.9)	0.000
HDL-C (mmol/L)	1.05 ± 0.36	1.32 ± 0.30	0.000
LDL-C (mmol/L)	2.53 ± 0.82	2.80 ± 0.72	0.000
TG (mmol/L)	1.48 ± 0.95	1.43 ± 1.08	0.524
TC (mmol/L)	4.30 ± 1.09	4.92 ± 1.43	0.000

^b^HDL-C, high-density lipoprotein cholesterol; LDL-C, low-density lipoprotein cholesterol; TG, triacylglycerol; TC, total cholesterol, P < 0.05 was statistically significant.

### The DSVs Identified in AMI Patients and Controls

**Figure 1:**
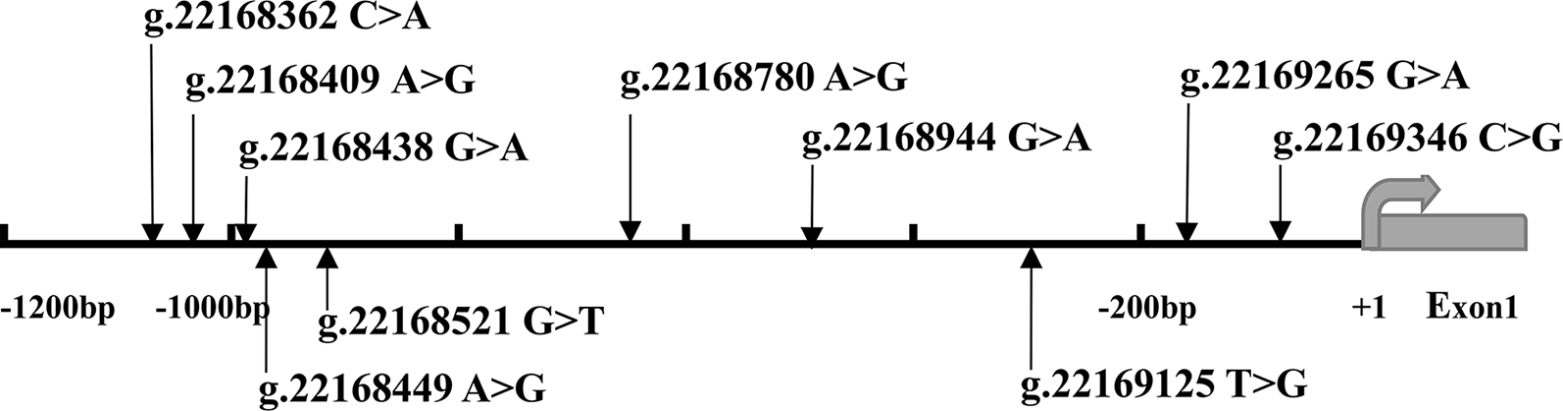
The DSVs and SNPs within the *GATA6* gene promoter identified in AMI patients and controls. Schematic representation of the identified *GATA6* gene DSVs. The DSVs were named according to their locations in the *GATA6* genomic sequences (NCBI: NC_000018.10). The transcription starts at 22169437 in the first exon that is untranslated.

**Figure 2:**
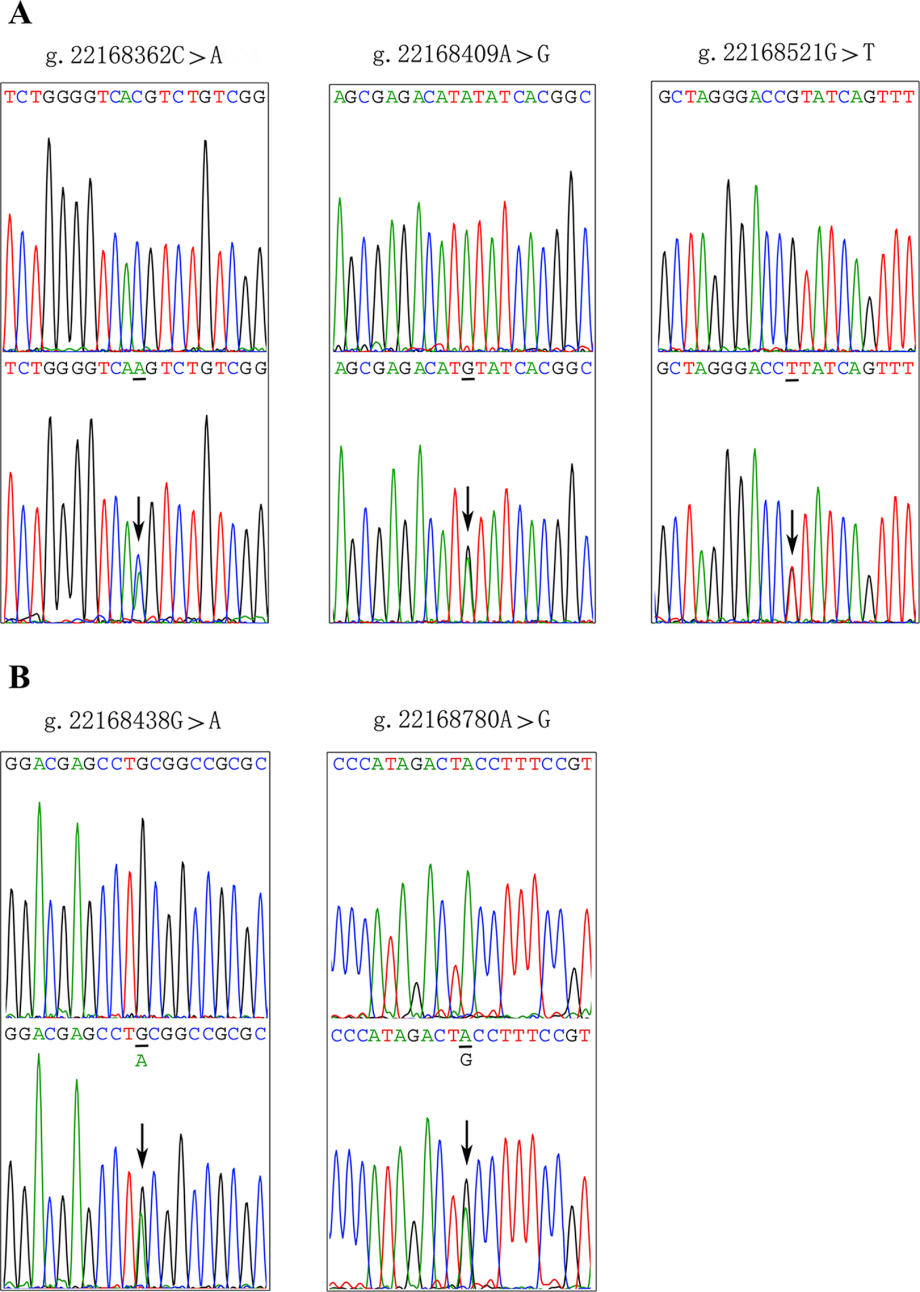
Sequencing chromatograms of the DSVs and SNPs within the *GATA6* gene promoter. **(A)**. Sequencing chromatograms of the DSV and SNPs in AMI patients. **(B)**. Sequencing chromatograms of the DSV and SNP in healthy controls. All sequence orientations of the DSVs and SNPs are forward. Top panels show wild type and bottom panels heterozygous DSVs or SNPs, which are marked with arrows.

After sequence detection of the *GATA6* gene promoter region by two overlapping PCR fragments, a total of 10 DSVs, including seven SNPs, were identified in 705 subjects. Its distribution had been summarized in **Table 3**. Meanwhile, gene positions of DSVs had also been shown in **Figure 1**, and oligonucleotide sequences of three DSVs were summarized in **Table 4**. Additionally, the summary of six SNPs for all participants can be seen in the **Supplemental Material** (http://review.frontiersin.org/review/474704/16/745502/#tab/History). One novel heterozygous DSV (g.22168409 A > G) and two SNPs [g.22168362 C > A(rs1416421760) and g.22168521 G > T(rs1445501474)] were identified in three AMI patients, which were not found in controls. It was worth noting that in this AMI patient with SNP [g.22168521 G > T(rs1445501474)], two other SNPs [g.22168944 G > A(rs144923558) and g.22169265 G > A(rs146748749)] were also identified. However, the latter two SNPs were also found in both other AMI patients and controls. The DNA sequencing chromatograms of these novel DSVs were shown in **Figure 2A**. Two novel heterozygous DSVs (g.22168438 G > A and g.22168780 A > G) were only found in two controls and the sequencing chromatograms of which were shown in **Figure 2B**. Especially, the location of novel heterozygous DSV (g.22168438 G > A) was also one SNP [g.22168438 G > T(rs958786414)], which was our new discovery. In addition, one novel heterozygous DSV (g.22169125 T > G) and four SNPs [g.22168449 A > G(rs189133474), g.22168944 G > A(rs144923558), g.22169265 G > A(rs146748749) and g.22169346 C > G(rs139399350)] were identified in both AMI patients and controls with similar frequencies (*P* > 0.05), the sequencing chromatograms of which were not shown.

**Table 3:** *GATA6* gene promoter DSVs in AMI patients and controls.

DSVs	Genotype	Location^c^	Controls (n = 353)	AMI (n = 352)	*P* value
g.22168362 C > A(rs1416421760)	CA	–1075bp	0	1	–
g.22168409 A > G	AG	–1028bp	0	1	–
g.22168438 G > A(rs958786414G/T)	GA	–999bp	1	0	–
g.22168449 A > G(rs189133474)	AG	–988bp	10	10	0.995
g.22168521 G > T(rs1445501474)	GT	–916bp	0	1	–
g.22168780 A > G	AG	–657bp	1	0	–
g.22168944 G > A(rs144923558)	GG	–493bp	335	334	1.000
	GA		18	17	
	AA		0	1	
g.22169125 T > G	TG	–312bp	1	1	1.000
g.22169265 G > A(rs146748749)	GG	–172bp	335	334	1.000
	GA		18	17	
	AA		0	1	–
g.22169346 C > G(rs139399350)	CC	–91bp	320	323	0.866
	CG		30	25	
	GG		3	3	

^c^DSVs were located upstream to the transcription start site of GATA6 gene at 22169437 of NC_000018.10.

**Table 4:** The double-stranded biotinylated oligonucleotides for the EMSA.

DSVs	Oligonucleotide	Loactions
g.22168362 C > A	5’-AGCCTTCTGGGGTCA(C/A)GTCTGTCGGAAGAAA-3’	22168347-22168377
g.22168409 A > G	5’-AAGGGAGCGAGACAT(A/G)TATCACGGCTCCTGC-3’	22168394-22168424
g.22168521 G > T	5’-TGCCCGCTAGGGACC(G/T)TATCAGTTTGTACAG-3’	22168506-22168536

### Association Between all SNPs in *GATA6* Gene Promoter and AMI Risk

In this study, six SNPs (rs1416421760, rs189133474, rs1445501474, rs144923558, rs146748749, rs139399350) were found in 352 AMI patients. The genotype and minimum allele frequencies (MAF) between AMI patients and controls were shown in **Table 5**. Based on the results of HWE analysis (*P* > 0.05), no data needs to be removed. The MAF of the six SNPs ranged from approximately 0.1% to 4.4% and 0% to 5.1% in AMI patients and controls, respectively. Unfortunately, no associations were observed between the six SNPs and AMI. Additionally, we assessed the association under five different genetic models (codominant, dominant, recessive, over-dominant, and log-additive) by the web-based software SNP-Stats. The result of analysis was shown in **Table 6**, in which none of genetic models in the SNPs of *GATA6* gene promoter was statistical significance with adjustment by age and sex (*P* > 0.05).

**Table 5:** Genotype and allele frequencies in six SNPs and odds ratio estimates for AMI risk.

SNP	Position	Alleles	AMI (n = 352)			HWE	Controls (n=353)			HWE	MAF		OR ( 95%CI )	*P v*alue
		(A/B)	AA	AB	BB	*P v*alue	AA	AB	BB	*P v*alue	AMI	Controls		
rs1416421760	22168362	A/C	0	1	351	0.978	0	0	353	1.000	0.001	0.000	–	–
rs189133474	22168449	G/A	0	10	342	0.787	0	10	343	0.787	0.014	0.014	0.99[0.41∼2.41]	0.994
rs1445501474	22168521	T/G	0	1	351	0.978	0	0	353	1.000	0.001	0.000	–	–
rs144923558	22168944	A/G	1	17	334	0.131	0	18	335	0.623	0.027	0.025	1.06[0.55∼2.03]	0.861
rs146748749	22169265	A/G	1	17	334	0.131	0	18	335	0.623	0.027	0.025	1.06[0.55∼2.03]	0.861
rs139399350	22169346	G/C	3	25	324	0.335	3	30	320	0.508	0.044	0.051	1.16[0.71∼1.91]	0.539

**Table 6:** Five genetic models’ analysis of the association between the SNPs in *GATA6* promoter region and AMI.

SNP ID	Model	Genotype	Control	AMI	OR(95%CI)	*P* value	AIC	BIC
rs144923558 or rs146748749	Codominant	G/C	335(94.9%)	334(94.9%)	1.00	0.98	677.3	700.1
		G/A	18(5.1%)	17(4.8%)	0.94(0.38-2.31)			
		A/A	0(0%)	1(0.3%)	–			
	Dominant	G/G	335(94.9%)	334(94.9%)	1.00	0.9	675.3	693.6
		G/A-A/A	18(5.1%)	18(5.1%)	0.94(0.38-2.32)			
	Recessive	G/G-G/A	353(100%)	351(99.7%)	1.00	0.86	675.3	693.5
		A/A	0(0%)	1(0.3%)	–			
	Over-dominant	G/G-A/A	335(94.9%)	335(95.2%)	1.00	0.89	675.3	693.5
		G/A	18(5.1%)	17(4.8%)	0.94(0.38-2.31)			
	Log-additive	–	–	–	0.95(0.39-2.31)	0.91	675.3	693.6
rs139399350	Codominant	C/C	320(90.7%)	324(92%)	1.00	0.28	674.8	697.6
			C/G	30(8.5%)	25(7.1%)	0.64(0.31-1.35)		
			G/G	3(0.8%)	3(0.8%)	0.33(0.05-2.23)		
	Dominant	C/C	320(90.7%)	324(92%)	1.00	0.14	673.2	691.4
			C/G-G/G	33(9.3%)	28(8%)	0.59(0.29-1.19)		
	Recessive	C/C-C/G	350(99.2%)	349(99.2%)	1.00	0.27	674.1	692.4
			G/G	3(0.8%)	3(0.8%)	–		
	Over-dominant	C/C-G/G	323(91.5%)	327(92.9%)	1.00	0.25	674	692.3
		C/G	30(8.5%)	25(7.1%)	0.65(0.31-1.36)			
	Log-additive	–	–	–	0.62(0.34-1.12)	0.11	672.8	691

### Associations Between Haplotype Analysis and AMI Risk

**Figure 3:**
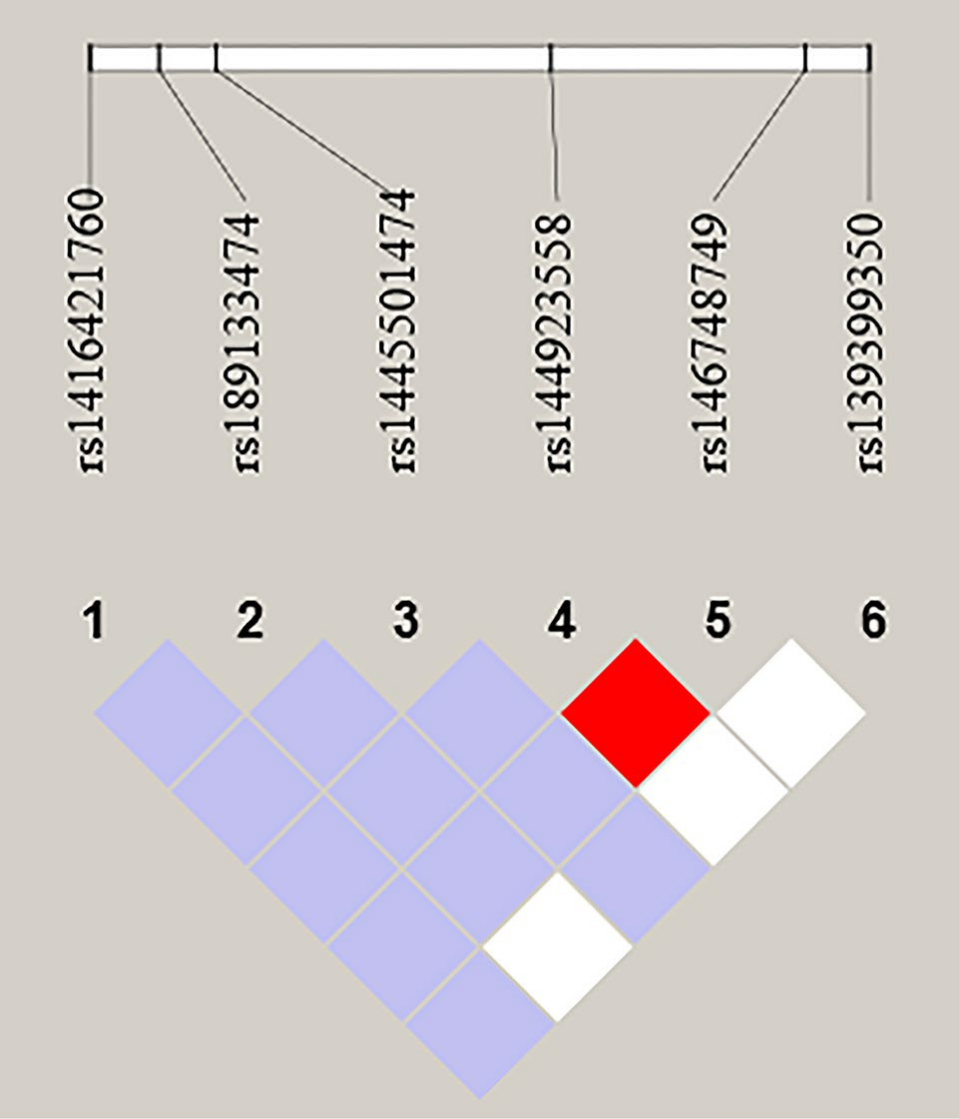
LD analysis of the six SNPs in AMI. Standard color schemes indicate different levels of LD. Bright red: LOD > 2, D' = 1; Purple: LOD < 2, D' < 1; White: LOD < 2, D' < 1; LOD: Log of the odds of there being LD between two loci.

We further performed LD and haplotype analysis for six SNPs by the HaploView software package (version 4.2) and the SHE-sis software platform. However, the only two candidate SNPs (rs144923558 and rs146748749) in *GATA6* gene promoter had showed strong linkage (D’ = 1.000, r^2^^=^ 1.000) (**Figure 3**).As were shown in **Table 7**, analysis results for the *GATA6* promoter region haplotypes were not found to be associated with a risk of AMI.

**Table 7:** Haplotype analysis of the SNPs in *GATA6* promoter region and the association with AMI risk^d^.

SNP1	SNP2	SNP3	SNP4	SNP5	SNP6	AMI(freq)	Control(freq)	Chi2	Fisher’s *P*	OR(95%CI)
C	A	G	A	A	C	18.00(0.026)	16.79(0.024)	0.044	0.833289	1.075(0.548-2.107)
C	A	G	A	A	G	0.00(0.00)	1.18(0.002)	1.175	0.278377	–
C	A	G	G	G	C	643.00(0.913)	646.87(0.916)	0.038	0.845477	0.963 [0.663∼1.400]
C	A	G	G	G	G	31.00(0.044)	30.13(0.044)	0.000	0.995988	0.999 [0.601∼1.661]
C	G	G	G	G	C	10.00(0.014)	6.30(0.009)	0.858	0.354305	1.599 [0.587∼4.357]
C	G	G	G	G	G	0.00(0.000)	3.70(0.005)	3.694	0.054652	–
A	A	G	G	G	C	1.00(0.001)	0.00(0.000)	1.003	0.316578	–
C	A	T	A	A	C	1.00(0.001)	0.00(0.000)	1.003	0.316578	–

^d^SNP (1∼6): rs1416421760, rs189133474, rs1445501474, rs144923558, rs146748749, rs139399350.

### SNP–SNP Interactions and AMI Risk

To verify the potential interactions between SNPs and the risk of AMI, the GMDR software package was used by us. In this study, the three best models were analyzed and **Table 8** summarized the cross‐validation consistency and the prediction error of the best models. However, the single model (rs139399350) had a maximum testing accuracy of 48.99% and a maximum cross‐validation consistency (6/10) that was still no significant at *P* < 0.05 level, after determined empirically by permutation testing.

**Table 8:** Predication of AMI risk factors in GMDR analysis.

Best model	Training accuracy(%)	Test accuracy(%)	Cross-validation consistency	Sign test	*P*-value
rs139399350	50.51	48.99	6/10	0	1.000
rs189133474, rs139399350	50.78	48.35	4/10	0	1.000
rs189133474, rs144923558, rs139399350	51.07	48.30	4/10	0	1.000

### A Power Statistical Calculation in This Case-Control Study

We computed the statistical power using a web browser program, Genetic Association Study Power Calculator (http://csg.sph.umich.edu/abecasis/gas_power_calculator/index.html), for this case-control study. The power calculation was conducted under various situations. For examples, the ratio of cases (n = 350) to controls (n = 350) was 1:1, and the significance level of the study design was set to 0.05. After analyzing the recent Chinese cardiovascular epidemiological reports, we set the disease prevalence to 0.10. The multiplication model was chosen for disease risk and the disease allele frequency was approximately 0.0043 (3/700). We assumed that Genotype Relative Risk was 1.5, 3.0, and 5.0 respectively. Besides, we selected “Cases+Controls” as the independent variable to plot against statistical power. Finally, the values of statistical power were 0.098, 0.506, and 0.896, respectively.

### DSVs-Related Putative Binding Sites for Transcription Factors

To further explore whether DSVs identified in the *GATA6* gene promoter of AMI patients affected the putative binding sites of other transcription factors, the JASPAR program (http://jaspar.genereg.net/) was fully utilized. As summarized in **Table 9**, the putative binding sites of the transcription factors could be abolished, modified, and created by DSVs identified in patients with AMI. Three binding sites for CAMP-response element binding protein (CREB1), sterol regulatory element binding transcription factor 2 (SREBF2), and upstream stimulatory factor 2 (USF2) may be abolished by the SNP [g.22168362C > A(rs1416421760)]. At the same time, it may create the binding site for SNAI2. The two binding sites for neurogenin 2 (NEUROG2) and Forkhead box L1 (FOXL1) may be abolished by the DSV (g.22168409 A > G), and the two binding sites for doublesex and mab-3 related 3 (DMRT3) and Forkhead box C1 (FOXC1) may also be created. In addition, the SNP [g.22168521 G > T(rs1445501474)] may abolish the binding sites for DMRT3 and FOXC1, and modify the binding site for GATA2, and create the binding sites for DUXA, GSC2, and GSX1.

**Table 9:** Predicted binding sites for transcription factors affected by the DSV and SNPs.

DSV/SNPs	Mode of action	Transcription factors
g.22168362C > A (rs1416421760)	abolish	CAMP-response element binding protein (CREB1); sterol regulatory element binding transcription factor 2 (SREBF2); upstream stimulatory factor 2 (USF2);
	create	snail family transcriptional repressor 2 (SNAI2)
g.22168409A > G	abolish	neurogenin 2 (NEUROG2); Forkhead box L1 (FOXL1);
	create	doublesex and mab-3 related 3 (DMRT3); Forkhead box C1 (FOXC1);
g.22168521G > T (rs1445501474)	abolish	doublesex and mab-3 related 3 (DMRT3); Forkhead box C1 (FOXC1);
	modify	GATA binding protein 2 (GATA2)
	create	double homeobox A (DUXA); goosecoid homeobox 2 (GSC2); GS homeobox 1 (GSX1);

### Functional Analysis of the DSVs by Dual-Luciferase Reporter Assay

To examine their transcriptional activities, wild-type, and variant *GATA6* gene promoters were cloned into luciferase reporter vector (pGL3-basic) to generate expression vectors, including empty pGL3-basic (negative control), pGL3-WT (wild-type *GATA6* gene promoter), pGL3-22168362A, pGL3-22168409G, pGL3-22168438A, pGL3-22168521T+22168944A+22169265A pGL3-22168521T, and pGL3-22168780G. It should be noted that pGL3-22168521T was a site-directed mutagenesis of pGL3-WT using Quick Mutation™ Site-Directed Mutagenesis Kit (Beyotime). These expression vectors were transfected into HEK-293 and H9c2 cells, respectively. After the predetermined time has elapsed, the cells were collected and assayed for dual luciferase activity. For better explanation of the results, the transcriptional activity of the wild-type *GATA6* gene promoter was set to 100%, and variant *GATA6* gene promoter transcriptional activities were compared with the wild-type.

**Figure 4:**
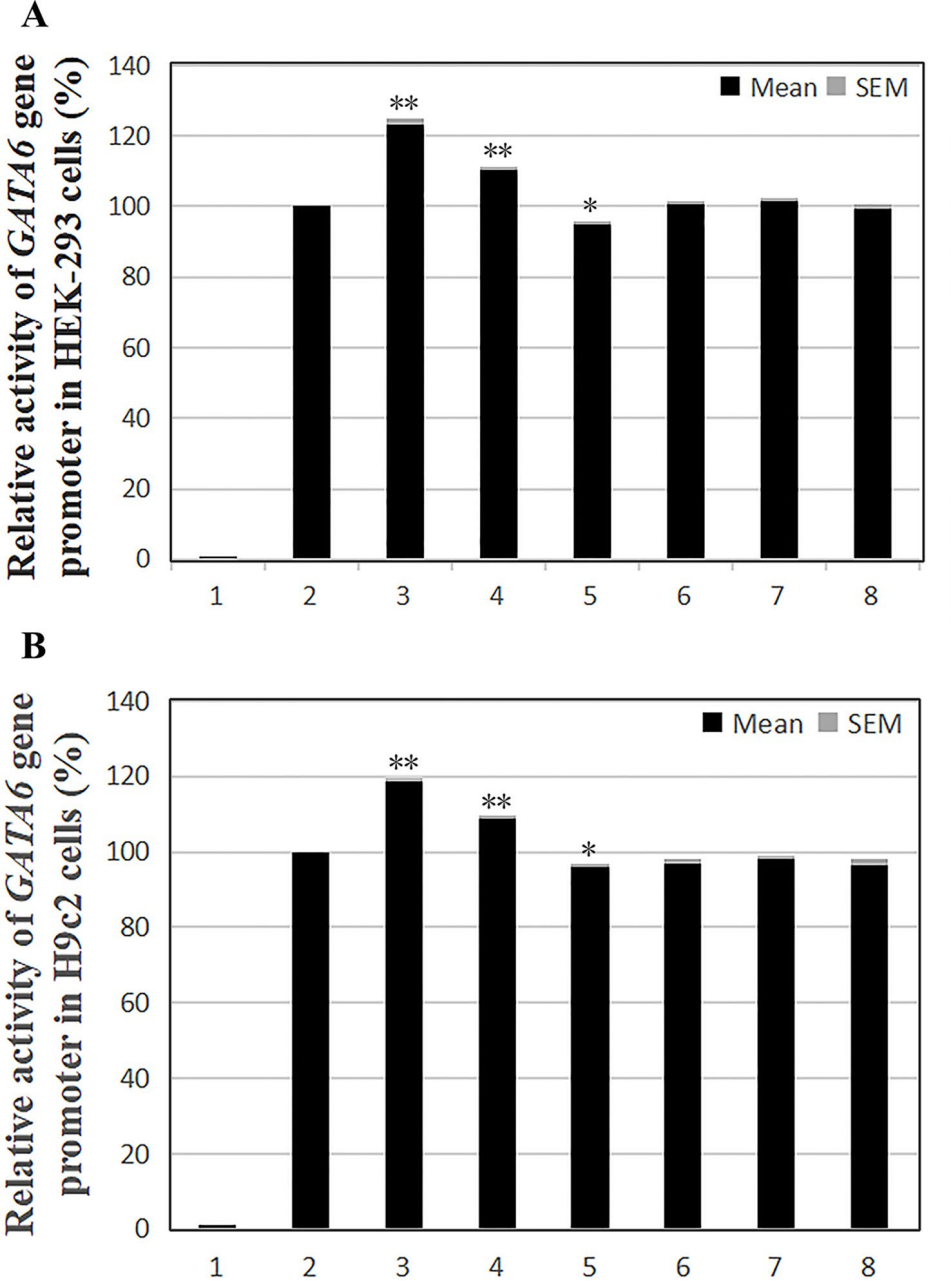
Relative transcriptional activity of wild-type and variant *GATA6* gene promoters. Wild-type and variant *GATA6* gene promoters were cloned into reporter gene vector pGL3 and transfected into cultured cells. The transfected cells were collected, and dual-luciferase activities were assayed. Empty vector pGL3-basic was used as a negative control. Transcriptional activity of the wild-type *GATA6* gene promoter was designed as 100%. Relative activities of *GATA6* gene promoters were calculated. **(A)** Relative activities of wild-type and variant *GATA6* gene promoters in HEK-293 cells. Lanes 1, pGL3-basic; 2, pGL3-WT; 3, pGL3-22168362A; 4, pGL3-22168409G; 5, pGL3-22168438A; 6, pGL3-22168521T+22168944A+22169265A; 7, pGL3-22168521T; 8, pGL3-22168780G. **(B)** Relative activities of wild-type and variant *GATA6* gene promoters in H9c2 cells. Lanes 1, pGL3-basic; 2, pGL3-WT; 3, pGL3-22168362A; 4, pGL3-22168409G; 5, pGL3-22168438A; 6, pGL3-22168521T+22168944A+22169265A; 7, pGL3-22168521T; 8, pGL3-22168780G. WT, wild-type. *, *P* 0.05; **, *P* 0.01.

According to the transfection results in HEK-293 cells, the transcriptional activity of the *GATA6* gene promoter was significantly increased by the novel heterozygous DSV (g.22168409 A > G) and SNP [g.22168362 C > A(rs1416421760)] identified in AMI patients (*P* < 0.01), and was not significantly altered by the SNP [g.22168521 G > T(rs1445501474)] which was also identified in patients with AMI (*P* > 0.05). On the other hand, the novel heterozygous DSV (g.22168780 A > G) only identified in controls did not alter the transcriptional activity of the *GATA6* gene promoter (*P* > 0.05). However, transcriptional activity of the *GATA6* gene promoter was significantly reduced by the SNP [g.22168438G > A (rs958786414G/T)] identified in controls (*P* < 0.05; **Figure 4A**). In addition, the transcriptional activities of wild-type and variant *GATA6* gene promoters were also examined in H9c2 cells. By comparing the transfection results of H9c2 cells with the results of HEK-293 cell transfection, it was found that the changes in the transcriptional activities of the wild-type and variant *GATA6* gene promoters were consistent in both cells. The novel heterozygous DSV (g.22168409 A > G) and the SNP [g.22168362 C > A(rs1416421760)] identified in AMI patients significantly increased transcriptional activities of the *GATA6* gene promoters (*P* < 0.01). As expected, in controls, the DSV (g.22168780 A > G) did not alter the transcriptional activity of the *GATA6* gene promoter (*P* > 0.05) and the SNP [g.22168438 G > A(rs958786414G/T)] significantly reduced transcriptional activity of the *GATA6* gene promoter (*P* < 0.05; **Figure 4B**).

In summary, the DSV/SNP [g.22168409 A > G and g.22168362 C > A (rs1416421760)] identified in AMI patients and the SNP [g.22168438 G > A(rs958786414G/T)] identified in controls significantly altered the transcriptional activity of the *GATA6* gene promoter in both HEK-293 cells and H9c2 cells, indicating the non-tissue specific effects of the DSV and SNPs on *GATA6* gene promoter.

### The Binding Sites for Transcription Factors Affected by the DSVs

**Figure 5:**
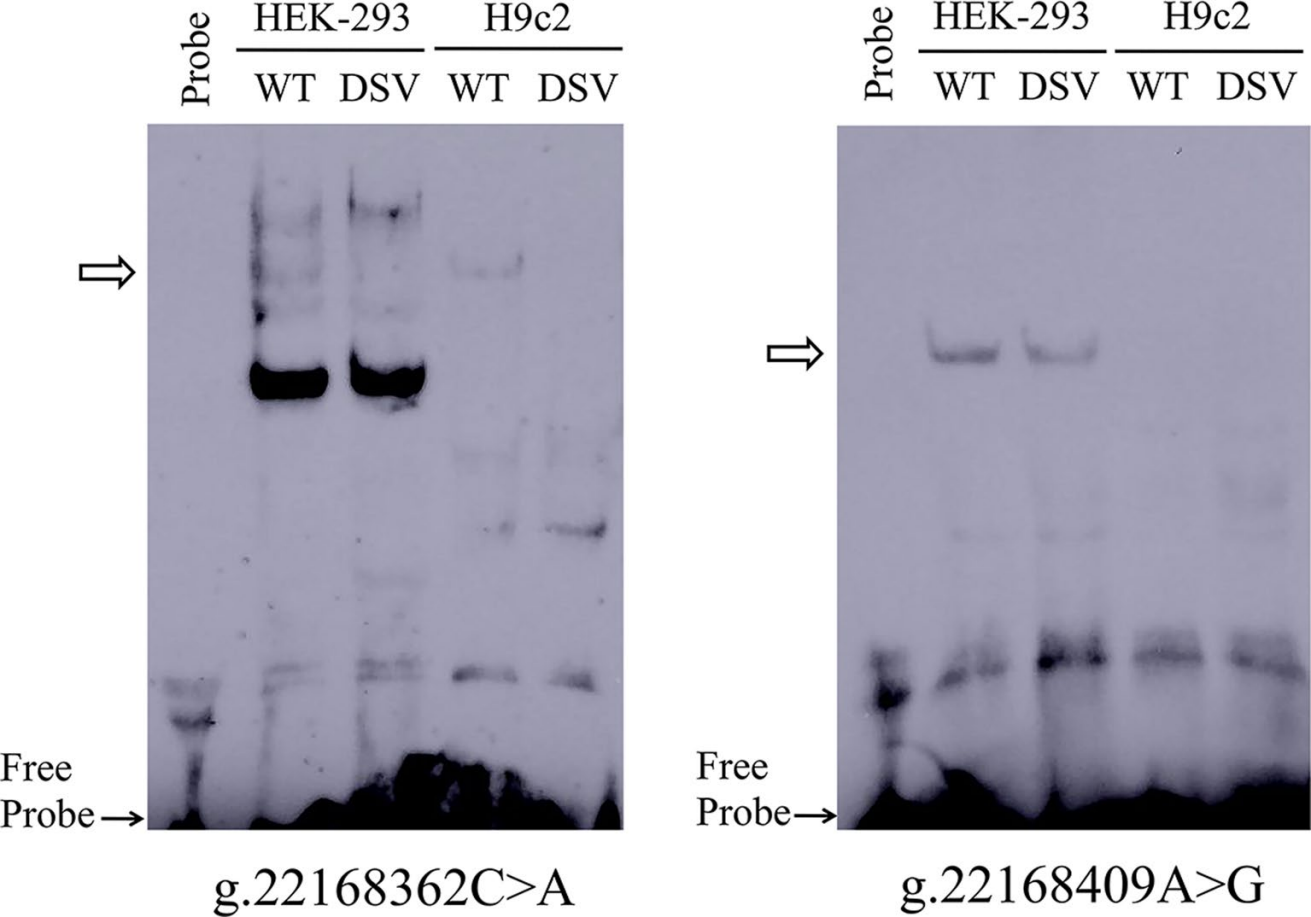
EMSA of biotin-labeled oligonucleotides containing DSV and SNP. Wild-type and variant oligonucleotides (31 bp) were designed and labeled with biotin for the DSV and SNP identified in AMI patients, including g.22168362C>A (rs1416421760) and g.22168409A>G. EMSA was conducted with biotinylated oligonucleotides and the nuclear extracts from HEK-293 and H9c2 cells. Free probe was marked with an arrow at the bottom. The affected binding for transcription factors was marked with an open arrow.

To further investigate whether the binding sites of some unknown transcription factors could be affected by the DSV and SNP identified only in AMI patients. EMSA was performed with wild-type and variant oligonucleotides. The sequences of the DSV and SNP had been shown in **Table 4**. The EMSA results showed that the SNP [g.22168362 C > A(rs1416421760)] reduced the binding of an unknown transcription factor in both HEK-293 and H9c2 cells. The DSV (g.22168409 A > G) reduced the binding of a transcription factor in HEK-293 cells and had no effect in H9c2 cells, which might be due to tissue cell specificity or sensitivity of EMSA experiments (**Figure 5**). Additionally, since the SNP [g.22168521 G > T(rs1445501474)] did not affect the *GATA6* gene promoter activity in cultured cells, this SNP was not tested with EMSA.

## Discussion

To date, no studies on genetic analysis of the *GATA6* gene promoter have been reported in CAD or AMI in adults. In our current study, genetic, and functional analysis of the *GATA6* gene promoter were performed in AMI patients and healthy controls from China. Although collective frequency of the novel DSV (g.22168409 A > G) and two SNPs [g.22168362 C > A(rs1416421760) and g.22168521 G > T(rs1445501474)] in *GATA6* gene promoter which were identified in AMI patients was 0.85% (3/352) and relevant statistical analysis, including allele and genotype frequencies between AMI patients and controls, five genetic models, LD analysis, and haplotype and SNP–SNP interactions, suggested no statistical significance (*P* > 0.05), the transcriptional activity of *GATA6* gene promoter was significantly increased by DSV (g.22168409 A > G) and SNP [g.22168362 C > A (rs1416421760)] in both HEK-293 and H9c2 cells *(P* < 0.01). Unexpectedly, the SNP [g.22168521 G > T(rs1445501474)] was identified in AMI patients, but did not significantly alter the transcriptional activity of the *GATA6* promoter (*P* > 0.05). In addition, the transcriptional activity of the *GATA6* gene was significantly decreased by the SNP [g.22168438 G> A(rs958786414G/T)] in both HEK-293 and H9c2 cells (*P* < 0.05). However, this SNP which was identified in controls and was not associated with the occurrence of AMI. Perhaps, it was associated with other human diseases that were still unknown. Furthermore, EMSA experiments revealed that the DSV (g.22168409 A > G) and the SNP [g.22168362 C > A(rs1416421760)] affected the binding of transcription factors. From these results of this study, we can conclude that the DSV (g.22168409A > G) and the SNP [g.22168362 C > A(rs1416421760)] may affect the binding of some unknown transcription factors and increase GATA6 levels in both HEK-293 and H9c2 cells.

Human *GATA6* gene is mapped to chromosome 18q11. 1 to q11. 2, consisting of seven exons and six introns spaced apart from each other. The whole process is 34 812 bp, encoding 595 amino acids, of which the first exon does not encode a protein (Suzuki et al., 1996). In human, *GATA6* gene is expressed in a variety of tissues. In addition to higher expression in fetal heart and lung, *GATA6* gene can also be expressed in adult heart, pancreas, ovary, lung, liver, central nervous system, adrenal, and VSMCs (Morrisey et al., 1996; Suzuki et al., 1996; Pikkarainen et al., 2004). Decreased or elevated expression of the *GATA6* gene has been reported to be associated with many cardiovascular diseases in humans, such as CHD, arrhythmia, cardiomyopathy, and neonatal and adult diabetes (Sun and Yan, 2019). The two *GATA6* gene DSVs identified in our study are located at -1,075 and -1,028 bp upstream of the transcription start site, which may affect the interaction with other transcription factors, enhancing their transcriptional activity. However, whether the change in GATA6 level is related to the occurrence and development of human AMI still requires further studies to verify.

AS occurs because vascular ECs and VSMCs are stimulated by various risk factors such as viruses, mechanical damage, immune complexes, especially ox-LDL, to cause an excessive chronic inflammatory proliferative reaction locally in the blood vessels (Gistera and Hansson, 2017; Katsiki et al., 2017; Pothineni et al., 2017). At the same time, a large number of genetic loci have been found to be associated with MI, CAD, thrombotic events, lipid traits, and atherothrombotic disease (Rader, 2015). Von Willebrand factor (VWF) is a molecule that is strictly specific for ECs and megakaryocytes (Liu et al., 2009). It mediates the adhesion of platelets to the endothelial and subendothelial surfaces, thus playing a major role in the initial stages of thrombosis (Springer, 2014). GATA6 may interact with other potential activators/co-activators and specifically bind to the *VWF* promoter in human ECs isolated from microvessels of the heart (cardiac MVEC) to enhance transcription (Mojiri et al., 2019). The mammalian target of rapamycin (mTOR) signaling has been reported to be associated with regulation of microRNAs (miRNAs) which are involved in diverse processes such as lipid metabolism and play fundamental roles in maintaining endothelial homeostasis and controlling leukocyte trafficking and inflammation, affecting the development of atherosclerosis (Novak et al., 2015; Fernandez-Hernando and Suarez, 2018). However, increase in protein kinase C α (PKCα) activity and miRNA-200a-3p expression caused by inhibition of mTOR independently decreased GATA6-mediated VCAM-1 expression and monocytic cell adhesion to human aortic endothelial cells (HAECs) monolayers (Fan et al., 2019). Additionally, it has been demonstrated that GATA motif is located in *VCAM-1* promoter region, and tumor necrosis factor-alpha (TNF-α) treatment resulted in the upregulation of GATA6 expression (Umetani et al., 2001). Phosphotase and tensin homolog (PTEN) which was a potent negative regulator of phosphatidylinositol-3-kinase (PI3K/Akt) pathway, selectively inhibited expression of VCAM-1 through modulation of PI3K/Akt/GSK-3/GATA-6 signaling cascade in TNF-α-treated ECs, causing significantly prevented monocyte adhesion to TNF-α-induced ECs (Tsoyi et al., 2010). It has been reported that in a unique CAD form called plaque erosion, VSMCs play a greater role in plaque formation than inflammatory cells (Arbustini et al., 1999). In a related animal experiment, mice with a CAD-linked LDL receptor-associated protein 6 (LRP6) mutation (LRP6R611C) showed coronary and aortic intimal hyperplasia even in the absence of mechanical vascular injury, which is mainly due to excessive VSMCs proliferation without excessive inflammation (Srivastava et al., 2015). As previously mentioned, the transcription factor GATA6 which is the predominant GATA family member expressed in VSMCs, cannot only promote VSMCs differentiation by stimulating the expression of contractile proteins and inhibiting cell cycle progression (Perlman et al., 1998), but also control the phenotype of these cells following vascular injury (Morrisey et al., 1996; Narita et al., 1996). Although GATA6 expression is downregulated in VSMCs following mitogen stimulation or vascular injury (Mano et al., 1999), under the action of stratified shear stress and urokinase plasminogen activator (a GATA6-dependent gene), GATA6 levels are elevated in ECs and elevated levels of GATA6 in atherosclerotic lesions (Sokabe et al., 2004). Furthermore, in the process of human atherosclerosis, Fas ligand (FasL) which is a 40-kDa cytotoxic type II transmembrane protein from the TNF family, is expressed together with markers of apoptosis in inflammatory regions of plaques (Geng et al., 1997; Martin-Ventura et al., 2004). FasL-mediated apoptosis of SMCs in fragile plaques may lead to plaque instability and rupture, which will promote MI (Geng et al., 1997; Kavurma et al., 2008). On the other hand, the plasma levels of soluble FasL are elevated in patients with AMI and unstable angina pectoris (Shimizu et al., 2002). Importantly, GATA6 is able to modulate SMCs apoptosis in an extracellular FasL-dependent manner, which regulates FasL transcription through -^298^TTATCA^-303^ (Tan et al., 2009). Moreover, Angiotensin II (Ang II) increases GATA6 and FasL expression and SMCs apoptosis in an Ang II type 2 (AT2) receptor-, caspase 8-, and extracellular FasL- dependent fashion (Tan et al., 2009).

In the past, it was thought that as the lesion of atherosclerosis increased, the lumen of the arteries narrowed, causing insufficient blood supply to tissues and organs, resulting in acute coronary syndromes (ACS) such as unstable angina, AMI, or sudden death. However, in recent years, it has been found that the occurrence of clinical symptoms after the formation of atherosclerotic lesions depends not only on the degree of stenosis of the arterial lumen, but also on whether the nature of the plaque itself is stable and whether there are secondary lesions such as thrombosis. As described above, increased transcriptional activity of the *GATA6* gene may promote specific binding of GATA6 to the promoter region of *VWF*, enhancing its transcriptional activity, and increase the risk of VWF-mediated thrombosis. Similarly, it is also possible to promote the expression of VCAM-1, causing adhesion of leukocytes in blood vessels. These factors can cause damage to the vascular endothelium. However, endothelial dysfunction leads to a decrease in the inhibition of platelet aggregation, which greatly weakens the body’s antithrombotic defense system, promotes the formation of blood clots, and eventually blocks the blood vessels and triggers AMI. In addition, increased expression of *GATA6* may affect the transcriptional activity of *FASL*, and promote the apoptosis of SMC, which may eventually lead to instability and rupture of intracoronary plaque, thereby promoting the occurrence of AMI.

Our study has several limitations. First, the small sample size was a major limitation in this study. The six variants we identified in patients with AMI did not show any statistical significance in multiple forms of comparative analysis. In addition, the value of statistical power was closely related to “Genotype Relative Risk”. Due to the limitation of the sample size, the value of the true “Genotype Relative Risk” had not yet been determined. Based on the data in this study, the value of statistical power can reach 0.8 or higher only when the Genotype Relative Risk is five or greater under the condition that the significance level of the study design is set to 0.05. Meanwhile, the value of the statistical power will increase as the sample size increases. Therefore, future studies need to be extended to thousands of samples to more accurately clarify whether there is a correlation between the *GATA6* gene promoter variants and the pathogenesis of AMI, or to identify rare variants of the *GATA6* gene promoter. Another limitation of this study was that we only selected the promoter region of the *GATA6* gene for study. The remaining regions of the *GATA6* gene still need to be explored in future studies, which is crucial for clarifying the relationship between the *GATA6* gene and AMI. Finally, we performed preliminary functional experiments on two variants identified in AMI patients using the HEK-293 and H9c2 cell lines. The high expression of the *GATA6* gene in human embryonic stage and the different sensitivity of experiments may be used as an explanation for the fact that the results seem stronger in HEK-293 cell line rather than the H9c2 cell line that is more cardiac related. Therefore, the conclusion that apply to humans still requires new evidences to support.

In conclusion, we were the first time to attempt genetic and functional variants analysis of the *GATA6* gene promoter in AMI patients and controls. A novel DSV (g.22168409 A > G) and a novel SNP [g.22168362 C> A (rs1416421760)] were identified in two AMI patients but not in controls. These two DSVs significantly increased the transcriptional activity of the *GATA6* gene promoter in both HEK-293 and H9c2 cell lines, and further EMSA showed that these two DSVs also significantly affected the binding of transcription factors. Whether the two variants identified in the *GATA6* gene promoter can promote the development and progression of human AMI by altering GATA6 levels still requires further studies to verify.

## Data Availability Statement

The datasets generated for this study can be found in the dbSNP, B153. Additionally, the raw data supporting the conclusions of this manuscript will be made available by the authors, without undue reservation, to any qualified researcher.

## Ethics Statement

The studies involving human participants were reviewed and approved by the Humanities and Ethics Committee of the Affiliated Hospital of Jining Medical University. The patients/participants provided their written informed consent to participate in this study.

## Author Contributions

ZS and BY conceived and designed the experiments; ZS and SP performed the experiments; ZS and YC analyzed the data; ZS and BY wrote the paper.

## Funding

National Natural Science Foundation of China, Grant numbers: 81370271, 81400291, 81670341, 81870279; Shandong Taishan Scholar Program, China, Grant number: tshw201502063.

## Conflict of Interest

The authors declare that the research was conducted in the absence of any commercial or financial relationships that could be construed as a potential conflict of interest.

## Supplementary Material

Supplementary Table 1 Summary of six SNPs for all participants.
